# Enfortumab vedotin induced interstitial lung disease: A first case report with pathological evidence from transbronchial lung cryobiopsy

**DOI:** 10.1016/j.rmcr.2025.102237

**Published:** 2025-05-22

**Authors:** Shota Kaburaki, Toru Tanaka, Koichiro Kamio, Yosuke Tanaka, Kazuo Kasahara, Yasuhiro Terasaki, Masahiro Seike

**Affiliations:** a Department of Pulmonary Medicine and Oncology, Graduate School of Medicine, Nippon Medical School, Tokyo, Japan; b Department of Analytic Human Pathology, Nippon Medical School, Tokyo, Japan

**Keywords:** Enfortumab vedotin, Interstitial lung disease, Antibody–drug conjugate, Nectin-4, Transbronchial cryobiopsy, Non-specific interstitial pneumonia

## Abstract

Enfortumab vedotin (EV), an antibody–drug conjugate targeting Nectin-4, has demonstrated efficacy against advanced urothelial carcinoma. While initially considered rare, EV-induced interstitial lung disease (ILD) is increasingly recognized, yet its pathological features remain poorly characterized. We report a case of a 60-year-old man with metastatic urothelial carcinoma who developed fever, fatigue, and cough after two cycles of EV therapy. His treatment history included right nephroureterectomy, platinum-based chemotherapy, and immune checkpoint inhibitors nivolumab and pembrolizumab. Laboratory tests revealed elevated serum ILD markers (Krebs von den Lungen-6527.7 U/mL, surfactant protein-D 294.5 ng/mL), and chest computed tomography showed new infiltrative shadows with air bronchogram predominantly in subpleural regions of the right lower lobe, consistent with organizing pneumonia pattern. Bronchoalveolar lavage from the right middle lobe showed 92 % macrophages with negative cultures. Transbronchial lung cryobiopsy revealed fibrosing nonspecific interstitial pneumonia with prominent fibrosis around bronchovascular bundles, lymphocytic infiltration in vessel walls and alveolar septa with myxofibrous thickening, epithelial injury, and fibrin exudation into alveolar spaces—representing previously undocumented features of EV-induced ILD. Drug discontinuation alone proved insufficient, but the patient improved markedly with methylprednisolone pulse therapy. This case highlights two key findings: detailed histopathological characterization through cryobiopsy documents distinct pathological features of EV-induced ILD; and early bronchoscopic evaluation helped guide therapeutic decision-making, supporting aggressive corticosteroid therapy. These findings advance our understanding of both the pathological features and management of EV-induced ILD, particularly relevant as EV–pembrolizumab combination becomes standard first-line treatment.

## 1 Introduction

Enfortumab vedotin (EV) is an antibody-drug conjugate (ADC) targeting Nectin-4 that has revolutionized the treatment landscape of advanced urothelial carcinoma. The pivotal phase III EV-301 trial demonstrated superior overall survival compared to chemotherapy (12.88 vs 8.97 months) in patients who progressed after platinum-based chemotherapy and immune checkpoint inhibitors [1]. While initially reported pulmonary toxicity was rare in clinical trials, real-world evidence has revealed concerning rates of interstitial lung disease (ILD). Recent retrospective analyses suggest that EV-induced pneumonitis may be more frequent than initially reported. In particular, Yoon et al. re-examined Korean patients enrolled in the EV-201 and EV-301 trials, observing pneumonitis in 28.1 % (18/64) of patients, with two fatalities [2]. Likewise, Desimpel et al. identified a 15 % rate of pleuro-pneumopathy in their real-world series of 20 patients, including symptomatic cases requiring high-dose steroids and one instance of successful EV rechallenge [3]. This observation warrants attention as EV-pembrolizumab combination was recently approved as first-line therapy for advanced urothelial carcinoma [4].

Despite the increasing recognition of EV-induced ILD, its pathological features remain poorly characterized. Most cases are diagnosed based on clinical and radiological findings alone, with limited understanding of the underlying histopathological changes. This knowledge gap is particularly concerning given the expanding use of EV, both as monotherapy and in combination with pembrolizumab [4]. Moreover, the distinction between EV-induced ILD and other causes of pulmonary toxicity, such as immune checkpoint inhibitor-induced pneumonitis, remains unclear in patients with prior immunotherapy exposure.

Here, we present a case of EV-induced ILD with detailed histopathological findings obtained through TBLC. The pathological evidence in this case provides unique insights into the mechanism of EV-induced ILD and demonstrates the potential value of early bronchoscopic evaluation in guiding therapeutic decisions, particularly in patients with complex treatment histories including prior immunotherapy.

## 2 Case presentation

A 60-year-old man with metastatic urothelial carcinoma presented with fever, fatigue, nausea, and cough. His cancer history included a transurethral resection of a bladder tumor for a papillary urothelial carcinoma (G1 pTa) of the left ureteral orifice approximately two years prior to the current presentation. Subsequently, approximately one year prior to the current presentation, he was diagnosed with right renal pelvic cancer (cT3N2M1). For the renal pelvic cancer, he received neoadjuvant chemotherapy with dose-dense methotrexate, vinblastine, doxorubicin, and cisplatin for 4 cycles. Eight months prior to the current presentation, he underwent open nephroureterectomy with lymph node dissection for the right renal pelvic cancer, revealing papillary urothelial carcinoma (predominantly Grade 3 with focal Grade 2 components, pT3, pN2) with lymphovascular invasion. He had a 35 pack-year smoking history but had quit 5 years ago. He had no other significant medical history and was not taking any medications other than chemotherapy agents.

Following surgery, he received 3 cycles of adjuvant nivolumab. Due to the development of multiple bone metastases, his treatment was changed to pembrolizumab, of which he completed 2 cycles. Two months before the presentation, he was started on EV at 1.25 mg/kg. On day 14 of his second cycle, he developed fever, fatigue, nausea, and cough. He was initially treated with levofloxacin for one week as an outpatient, but due to lack of improvement, he was admitted for further evaluation. His pulmonary function tests at the time revealed forced vital capacity (FVC) of 3.37 L (96.6 % predicted), forced expiratory volume in 1 s (FEV_1_) of 2.62 L (94.9 % predicted), FEV_1_/FVC ratio of 77.7 %, and diffusing capacity for carbon monoxide of 11.95 (65.7 % predicted).

**Fig. 1:**
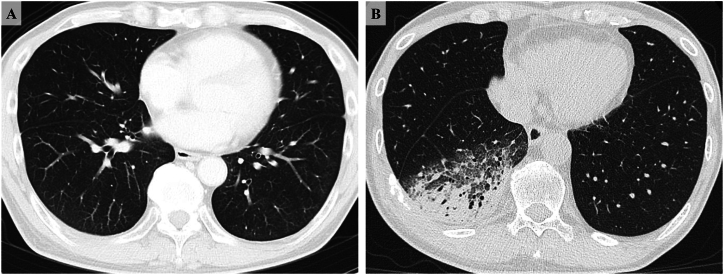
High-Resolution Computed Tomography (HRCT) Findings. (A) Baseline HRCT obtained prior to enfortumab vedotin initiation, showing no abnormal findings. (B) HRCT at presentation, revealing newly developed infiltrative shadows with air bronchograms predominantly in the right lower lobe. The peribronchovascular distribution of the infiltrates and subtle interlobular septal thickening in the periphery are consistent with an organizing pneumonia pattern.

On physical examination, his vital signs showed a temperature of 38.5 °C, and oxygen saturation of 96 % on room air. Laboratory tests showed a white blood cell count of 7100/μL and C-reactive protein level of 13.0 mg/dL. Serum markers for ILD were elevated, with Krebs von den Lungen-6 level of 527.7 U/mL and surfactant protein-D level of 294.5 ng/mL. Chest high-resolution computed tomography (HRCT) revealed newly developed infiltrative shadows with air bronchogram predominantly in subpleural regions of the right lower lobe, consistent with organizing pneumonia (OP) pattern, which were absent in the baseline images obtained before EV initiation (Fig. 1). Echocardiography showed no abnormal findings. Tests for influenza and severe acute respiratory syndrome coronavirus 2 antigens were negative.

**Fig. 2:**
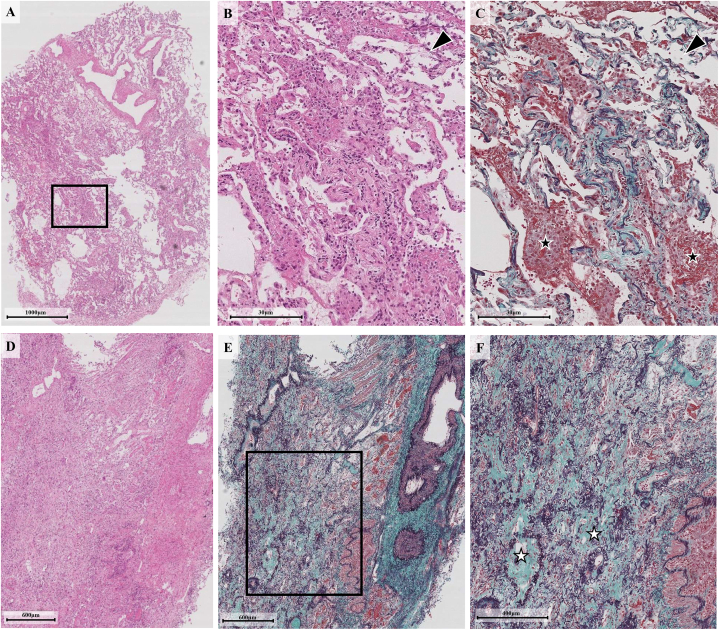
Histopathological Findings from Transbronchial Lung Cryobiopsy (TBLC). Panels (A) through (C) show specimens from right B^9^a. In (A), a low-magnification hematoxylin and eosin (H&E)-stained TBLC specimen demonstrates a diffuse fibrosing nonspecific interstitial pneumonia pattern with cellular features. The boxed area in (A) is magnified in (B) and (C). In (B), a higher-magnification H&E image shows mild inflammatory cell infiltration and focal areas of epithelial injury with denudation of the alveolar surface (arrowhead). In (C), an Elastica Masson-Goldner (EMG) stain highlights fibrin deposition within the alveolar spaces (black stars), without the hyaline membranes typically seen in diffuse alveolar damage. Panels (D) through (F) show specimens from right B^9^b. In (D), a low-magnification H&E view reveals a similar pattern of interstitial inflammation, while (E), a low-magnification EMG view of the same specimen, shows relatively preserved underlying lung architecture. In (F), a higher-magnification EMG image of the boxed area in (E) demonstrates prominent obliterative fibrosis within small airways and alveolar ducts (white stars), consistent with obliterative intraluminal fibrosis.

Bronchoalveolar lavage analysis from the right middle lobe, which showed no radiographic abnormalities, showed 92 % macrophages, 4 % lymphocytes, and 4 % neutrophils with a CD4/CD8 ratio of 1.0. Cultures for bacteria, fungi, and acid-fast bacilli, as well as multiplex respiratory viral assays of the lavage fluid were all negative. TBLC revealed fibrosing nonspecific interstitial pneumonia (NSIP) pattern with prominent fibrosis around the bronchovascular bundles and veins. The specimen showed lymphocytic infiltration in the vessel walls and alveolar septa with myxofibrous thickening, and fibrin exudation into the alveolar spaces (Fig. 2). Given these findings and clinical presentation, the patient was diagnosed with EV-induced ILD. Although EV was discontinued, his condition did not improve with supportive care alone. He was treated with methylprednisolone pulse therapy (500 mg/day), which resulted in gradual improvement of his symptoms and radiological findings. He was discharged on oral prednisolone 30 mg daily.

## 3 Discussion

This case highlights two significant findings regarding EV-induced ILD. First, the TBLC revealed a fibrosing NSIP pattern with prominent epithelial injury and fibrin exudation, providing novel pathological insights into EV-induced ILD. Second, while drug discontinuation alone was insufficient, early recognition of these pathological features guided the decision for aggressive corticosteroid therapy, leading to clinical improvement. These findings advance our understanding of both the pathological features of EV-induced ILD and its optimal management strategy.

The pathological findings in our case revealed unique features that may contribute to understanding EV-induced ILD. While the HRCT showed an OP pattern, histopathological examination demonstrated unexpected epithelial injury with fibrin exudation. This pattern differs from the predominant OP pattern reported in immune checkpoint inhibitor-induced pneumonitis, though the contribution of prior immunotherapy cannot be completely excluded in our case [5]. Several potential mechanisms could explain these findings. While Nectin-4 expression is generally low in normal lung tissue, inflammation and tissue injury may induce local changes in expression or facilitate off-target uptake of the ADC [6]. The observed epithelial injury, in particular, suggests a possible role for the direct cytotoxic effects of the monomethyl auristatin E (MMAE) payload. Preclinical work by Challita-Eid et al. established that enfortumab vedotin's cytotoxic activity is primarily mediated by this MMAE payload, which can exert potent cytotoxic effects not only in Nectin-4-expressing tumor cells but also potentially in non-tumoral tissues, possibly via target-independent mechanisms or off-target uptake, as indicated by studies on ADCs [7]. In particular, recent data indicate that EV can exhibit high cytotoxicity in normal tissues such as the skin and lungs, presumably via non-specific uptake of the MMAE payload [8]. Furthermore, the human IgG1 backbone of EV may activate complement and engage Fc receptor-bearing immune cells, leading to localized inflammatory responses and tissue damage [7]. Similarly, Swain et al. reported that ADC like trastuzumab deruxtecan can trigger ILD through both immune-mediated and off-target pathways, underscoring the importance of proactive pulmonary monitoring [9]. The presence of acute epithelial damage in addition to fibrosing NSIP suggests a potentially more severe form of ILD, which might explain the rapid clinical deterioration seen in some cases [2]. Understanding these multiple potential mechanisms will be crucial as EV use expands, particularly in combination with immunotherapy.

Our bronchoscopic evaluation, including TBLC, contributed to the management of EV-induced ILD in this case. While tissue confirmation is not considered mandatory for drug-induced ILD diagnosis, bronchoscopic evaluation can help confirm the presence of interstitial pneumonia and exclude infection, potentially supporting earlier diagnosis and treatment decisions [10,11]. Though bronchoalveolar lavage analysis, particularly lymphocyte fractions, might suggest potential steroid responsiveness, previous studies have indicated that lavage cellular profiles may not accurately reflect the true extent of inflammatory changes in the lung parenchyma [12,13]. TBLC is considered a relatively safe procedure with a diagnostic yield of approximately 80 % in ILD [14]. While most EV-induced ILD cases can be appropriately managed based on clinical and radiological findings alone, bronchoscopic evaluation helped exclude infection and provided additional insights through histopathological findings [2]. The OP pattern observed on HRCT in our case was consistent with previously reported radiological findings of EV-induced ILD [2]. However, the presence of acute epithelial injury despite OPpattern on imaging supported our decision to initiate high-dose corticosteroids rather than observation or drug discontinuation alone. While tissue diagnosis is not mandatory for drug-induced ILD management, it may provide useful information in selected cases, particularly in patients with complex treatment histories including prior immunotherapy, where the cause of lung toxicity can be unclear.

A key consideration in this case was distinguishing EV-induced ILD from both idiopathic NSIP (iNSIP) and immune checkpoint inhibitor (ICI)-induced ILD, given the patient's prior treatment with nivolumab and pembrolizumab. Several features support EV-induced ILD as the most likely diagnosis. First, the temporal relationship between symptom onset, occurring 14 days after the second cycle of EV, aligns with previously reported timelines for EV-induced ILD [2]. Second, although the histopathological findings demonstrated an NSIP pattern, the presence of prominent epithelial injury, fibrin exudation, and obliterative intraluminal fibrosis within small airways and alveolar ducts is less common in iNSIP yet has been described in drug-induced ILD [10]. Regarding ICI-induced ILD, the interval between the last ICI dose and the onset of pneumonitis in this patient (approximately two months) exceeds the more commonly reported timeframe [5]. Finally, the patient's marked and rapid improvement with corticosteroid therapy, despite the presence of fibrosis, also supports a recent drug-induced process rather than an idiopathic chronic interstitial pneumonia [15].

The management of EV-induced ILD requires a tailored approach based on disease severity and timing of recognition. Early bronchoscopic evaluation may identify patients requiring aggressive intervention, as demonstrated in our case where pathological findings supported the use of high-dose corticosteroids. Given the increasing adoption of EV, both as monotherapy and in combination with pembrolizumab, establishing standardized monitoring and management protocols becomes crucial [4]. Furthermore, prior exposure to ICIs may amplify the inflammatory milieu in the lung, potentially enhancing EV-related toxicity. Heath & Rosenberg suggest that EV's immunogenic effects, such as upregulating major histocompatibility complex genes, might synergize with residual immune activation from prior ICI therapy, increasing the risk of pneumonitis [6]. Regular radiological screening and prompt evaluation of respiratory symptoms may help identify lung toxicity before severe clinical deterioration occurs. Although some patients may tolerate EV at a reduced dose or with concurrent corticosteroids, further prospective data are needed to define best practices for rechallenge after severe pneumopathy.

In summary, our case, together with emerging evidence from recent reports, underscores the importance of vigilant monitoring for EV-induced ILD. Mechanistic studies suggest that off-target toxicity, Nectin-4 expression changes, and interplay with prior immunotherapy might drive pulmonary complications. Larger prospective and registry-based investigations are warranted to clarify risk factors, optimize diagnostic protocols, and develop standardized management algorithms, including guidance on EV rechallenge after ILD.

## CRediT authorship contribution statement

**Shota Kaburaki:** Writing – original draft, Resources, Investigation, Conceptualization. **Toru Tanaka:** Writing – review & editing, Supervision, Investigation. **Koichiro Kamio:** Writing – review & editing, Supervision, Investigation. **Yosuke Tanaka:** Writing – review & editing, Investigation. **Kazuo Kasahara:** Writing – review & editing, Investigation. **Yasuhiro Terasaki:** Writing – review & editing, Supervision, Investigation. **Masahiro Seike:** Writing – review & editing, Writing – original draft, Supervision, Resources, Investigation.

## Ethical approval

Given that this was a single case report, ethics committee approval was not needed.

## Clinical trial number

Not applicable.

## Consent for publication

Written informed consent was obtained from the patient for publication of this case report and accompanying images.

## Availability of data and materials

Not applicable.

## Funding

No funding or sponsorship was received for this study or publication of this article.

## Declaration of competing interest

The authors declare that they have no known competing financial interests or personal relationships that could have appeared to influence the work reported in this paper.
